# ORR in Non-Aqueous Solvent for Li-Air Batteries: The Influence of Doped MnO_2_-Nanoelectrocatalyst

**DOI:** 10.3390/nano10091735

**Published:** 2020-09-01

**Authors:** Eleonora Pargoletti, Annalisa Salvi, Alessia Giordana, Giuseppina Cerrato, Mariangela Longhi, Alessandro Minguzzi, Giuseppe Cappelletti, Alberto Vertova

**Affiliations:** 1 Dipartimento di Chimica, Università degli Studi di Milano, Via Golgi 19, 20133 Milan, Italy; eleonora.pargoletti@unimi.it (E.P.); annalisa.salvi@studenti.unimi.it (A.S.); mariangela.longhi@unimi.it (M.L.); alessandro.minguzzi@unimi.it (A.M.); 2 Consorzio Interuniversitario per la Scienza e Tecnologia dei Materiali (INSTM), Via Giusti 9, 50121 Firenze, Italy; 3 Dipartimento di Chimica & NIS, Università degli Studi di Torino, Via P. Giuria 7, 10125 Turin, Italy; alessia.giordana@unito.it (A.G.); giuseppina.cerrato@unito.it (G.C.)

**Keywords:** manganese dioxide nanoparticles, cobalt doping, iron doping, electrocatalyst, oxygen reduction reaction, organic solvent, Li-air battery

## Abstract

One of the major drawbacks in Lithium-air batteries is the sluggish kinetics of the oxygen reduction reaction (ORR). In this context, better performances can be achieved by adopting a suitable electrocatalyst, such as MnO_2_. Herein, we tried to design nano-MnO_2_ tuning the final ORR electroactivity by tailoring the doping agent (Co or Fe) and its content (2% or 5% molar ratios). Staircase-linear sweep voltammetries (S-LSV) were performed to investigate the nanopowders electrocatalytic behavior in organic solvent (propylene carbonate, PC and 0.15 M LiNO_3_ as electrolyte). Two percent Co-doped MnO_2_ revealed to be the best-performing sample in terms of ORR onset shift (of ~130 mV with respect to bare glassy carbon electrode), due to its great lattice defectivity and presence of the highly electroactive γ polymorph (by X-ray diffraction analyses, XRPD and infrared spectroscopy, FTIR). 5% Co together with 2% Fe could also be promising, since they exhibited fewer diffusive limitations, mainly due to their peculiar pore distribution (by Brunauer–Emmett-Teller, BET) that disfavored the cathode clogging. Particularly, a too-high Fe content led to iron segregation (by energy dispersive X-ray spectroscopy, EDX, X-ray photoelectron spectroscopy, XPS and FTIR) provoking a decrease of the electroactive sites, with negative consequences for the ORR.

## 1. Introduction

In the last few years, rechargeable metal-air batteries (MABs, as lithium-air or zinc-air ones) have gained renovate attention due to their feasibility as both electrochemical energy storage and conversion devices [1,2]. Indeed, due to their potentially very high theoretical energy density (around 3600 Wh kg^−1^, which is almost eight-fold times the value reported for Li-ion cells [1,3]), they can be considered as one of the most promising technologies in the energetic field. However, their engineering and commercialization have been significantly hindered by their scarce cycle life because of the sluggish kinetics of either the cathodic oxygen reduction reaction (ORR) or the anodic oxygen evolution reaction (OER) [4,5]. Specifically, focusing on the Li-air battery discharge process, molecular oxygen can be reduced to oxygen superoxide, peroxide or oxide, thus forming the corresponding LiO_2_, Li_2_O_2_ or Li_2_O species [3,6,7]. Especially the latter insoluble compound can cause the formation of lithium dendrites that clog the active sites impeding the oxygen diffusion and raising battery safety issues, such as thermal instability, internal short-circuits and huge charging overpotentials [2,3]. Hence, the fabrication of highly efficient ORR and OER catalysts is of paramount importance to improve the cyclic stability and longevity of these devices [2], since these materials must both facilitate the decomposition of LiO_2_/Li_2_O_2_ and control the side reactions.

An optimal bifunctional electrocatalyst should comprise high electrical conductivity, specific surface area and great porosity [8,9]. Among the widely exploited nanomaterials, Pt/C, RuO_2_ and IrO_2_ revealed to be the best candidate to be used in metal-air devices, notwithstanding their high costs [2,10,11,12]. For this reason, in the last decades, the research attention has been focused on the exploitation of cheaper transition metal oxides as MnO_2_ [9,13,14], Co_3_O_4_ [15], Fe_3_O_4_ [16,17], and so on. Among them, manganese oxides were found to be promising because of the abundance of manganese, low cost, scarce toxicity, relative high activity and presence of several valence states/polymorphic phases [18,19,20]. Moreover, MnO_2_ has been extensively studied for supercapacitor applications due to its high specific capacitance [18]. Nevertheless, the already reported performances of manganese dioxide nano-electrocatalysts rarely achieve the well-performing Pt- or Ru-based materials [21]. Therefore, recent studies were devoted to enhancing its electrochemical features by coupling manganese dioxide with conductive additives like carbon nanotubes [22], polymers [23], graphene [24] and/or by doping with various transition metals (such as Ni [25], V [21], Ce [25], Co [26] and Fe [19]) to improve the electron transport features. For instance, Kim et al. [26] reported the 5% Co-doped MnO_2_ nanoparticles exhibiting an excellent capacitance retention (of about 97%) after 5000 charge/discharge cycles. Additionally, some authors of the present paper have recently proved that the 1% Ag-doping of manganese dioxide lattice allows reaching very high specific capacity close to 1400 mAh g^−1^, with a considerable high charge retention through cycles [27]. However, most of these studies concerning the exploitation of differently modified MnO_2_ nanoparticles were carried out in aqueous solvent. Conversely, our aim is to identify and investigate potential electrocatalysts to be applied in lithium–air devices, i.e. in non-aqueous electrolytes widely used to prevent Li decomposition. Nonetheless, as largely reported in literature [28,29], the organic solvents may be affected by electrode surface potentials, causing a rapid degradation of the electrolyte itself and resulting in the formation of other discharge products (lithium alkyl carbonates or simply Li_2_CO_3_) [28,30]. The usage of propylene carbonate (PC)—and in general of organic carbonates—is still an open debate and studies on the mechanism and byproducts formation are still going on [31]. To overcome this problem, aprotic electrolytes (such as dimethyl sulfoxide, DMSO or tetraethylene glycol dimethyl ether (TEGDME)) have been recently used in Li-O_2_ batteries [13,32,33]. However, also these newly adopted solvents have some shortcomings, as undesired reactions leading to the formation of Li_2_CO_3_ and LiOH [34,35]. Therefore, an optimal solvent does not exist so far: for this reason, in the present work, we adopted PC as solvent and LiNO_3_ (0.15 M) as electrolyte, due to both their marketability and the PC very high efficiency in stabilizing the intermediate LiO_2_, thus favoring the formation of Li_2_O_2_ rather than Li_2_O [13,36].

Hence, we report here the hydrothermal synthesis of both bare and differently doped-MnO_2_ nanoparticles to be used as efficient electrocatalysts for the ORR in lithium-air batteries. Cobalt and iron ions with two different molar percentages (2% and 5%) were incorporated into the MnO_2_ lattice, resulting in an ORR enhancement especially for the cobalt-doped nanomaterials. Notably, both structural, surface and morphologic features were deeply tuned by tailoring the doping agent and its content. Therefore, a detailed correlation between their electrocatalytic behavior and physicochemical properties was finely drawn.

## 2. Materials and Methods

All the chemicals were of reagent-grade purity and were used without further purification. Doubly distilled water passed through a MilliQ apparatus (Merck KGaA, Darmstadt, Germany) was utilized.

### 2.1. Synthesis of Bare and Co-/Fe-Doped Nano-MnO_2_

Bare and doped samples were synthesized through hydrothermal route. As concerns the synthesis of pure MnO_2_, the appropriate amount of manganese acetate salt precursor (32 mmol Mn(CH_3_COO)_2_ × 4H_2_O, purchased by Sigma-Aldrich, St. Louis, MO, USA; 99.99% purity) was dissolved in 30 cm^3^ of 5% ethanol–water solution, under vigorous stirring at 80 °C. Then, 32 mmol of the oxidizing agent (NH_4_)_2_S_2_O_8_ (purchased by Sigma-Aldrich, St. Louis, MO, USA; 98% reagent grade), dissolved in other 30 cm^3^ of 5% ethanol-water, were added dropwise to the previous solution. The system was kept under stirring for 7 h. Subsequently, it was cooled down at room temperature and left to settle for a night. After the reaction was completed, the resulting brownish-black solid product was centrifuged and washed several times with MilliQ water, until the pH became neutral. Then, the product was dried in oven at 60 °C for about 24 h.

An analogous procedure was adopted for the Co-/Fe-doped MnO_2_ powders, adding the dopant precursors (Co(NO_3_)_2_ or Fe(NO_3_)_2_, respectively) in order to have dopant/manganese molar ratios equal to 2% and 5%, for each doping agent.

All samples were named as nx_MnO_2_, where n is the doping percentage (2% or 5%) while x stands for Co or Fe.

### 2.2. Powders Physicochemical Characterization

X-ray powder diffraction (XRPD) analyses were performed on a Rigaku (Tokyo, Japan) MiniFlex 600 Bragg–Brentano goniometer. We used graphite-monochromated Cu K_α_ radiation (Cu K_α1_ *λ* = 1.54056 Å, K_α2_ *λ* = 1.54433 Å); diffraction patterns were collected between 5° and 80° with a step size of 0.1°.

Mid and far infrared spectroscopy (FIR) spectra were recorded in the range 4000–0 cm^−1^ using a Bruker (Billerica, MA, USA) Vertex 70 spectrophotometer, equipped with Harrick MVP2 ATR cell and DTGS detectors. The adopted resolution was equal to 4 cm^−1^.

Specific surface area and porosity distribution were determined from N_2_ adsorption/desorption isotherms at 77 K using a Micromeritics Tristar II 3020 (Norcross, GA, USA) apparatus and the instrumental software (Version 1.03, GA, USA) by applying Brunauer-Emmett-Teller (BET) and Barrett-Joyner-Halenda (BJH) analyses, respectively. Before measurements, sample powders were pretreated at T = 150 °C (4 h under N_2_ flux) to remove adsorbed species.

FEG-SEM images were obtained by means of a TESCAN (Brno, Czech Republic) S9000G microscope, equipped with a Schottky emitter source at a resolution of 0.7 nm at 15 keV (In-Beam SE and a EDX spectrometer by OXFORD (Detector Ultim Max and software AZTEC).

X-ray photoelectron spectroscopy (XPS) (Thermo Scientific, MA, USA) analysis was carried out by means of an M-probe apparatus (Thermo Scientific, MA, USA), using a monochromatic Al K_α_ radiation source (1486.6 eV). The XPS binding energy scale was charge corrected using the standard calibration, fixing the C 1s peak at 284.6 eV.

### 2.3. Electrochemical Characterization

For the electrochemical characterization, we exploited a three-electrodes conventional cell based on a working MnO_2_ powder modified-glassy carbon electrode (MnO_2_-GCE, W), a ferrocene quasi-reference electrode (Fc/Fc^+^, R) and a Pt foil as the counter electrode (C). MnO_2_-GCEs were prepared by drop-casting 15 µL of a slurry made of 0.2 mg of catalyst and 0.8 mg of carbon vulcan XC-72R (Cabot^®^) dispersed in 1 mL of 2-propanol, to which 9.5 µL of a solution containing 0.15% wt of Tokuyama in 2-propanol was added. The slurry was sonicated for about 5 hours in order to obtain a homogeneous suspension. Before carrying out the electrochemical tests, solvent was let to completely evaporate in the air. The cell was assembled in glovebox under argon inert atmosphere. Electrochemical tests were carried out using propylene carbonate (PC)/0.15 M LiNO_3_ electrolyte. Furthermore, unless otherwise stated, all the potentials will be rescaled to the Li/Li^+^ potential. High-purity (99.9995%) oxygen was fed directly inside the three-electrodes conventional cell, both before (for 20 min) and during the electrochemical test (a bubbler was used to control the gas flux). The cell was studied by performing staircase–linear sweep voltammetries (S-LSVs) in the potential range between 3.04 and 1.64 V (vs. Li/Li^+^) and by evaluating the onset potential for the ORR, through the first derivative of the recorded S-LSVs. Current density values were reported considering the geometric GCE area equal to 0.159 cm^2^. S-LSVs were registered with a scan rate of 5 mV s^−1^, recording the current at the end of each potential step, while the electrode rotation frequency was set to 1000 rpm to support the reactant transport to and from the electrode surface. Moreover, in order to have a blank reference the same electrochemical tests were conducted either in an inert N_2_ atmosphere or by using bare glassy carbon electrode (GCE).

An Autolab PGSTAT204 potentiostat/galvanostat was employed to perform S-LSV tests and Nova 2.1.4 software (Metrhom, Herisau, Swiss) was used for data acquisition.

## 3. Results

### 3.1. Co- and Fe-Doped MnO_2_ Electrocatalytic Behavior

**Figure 1:**
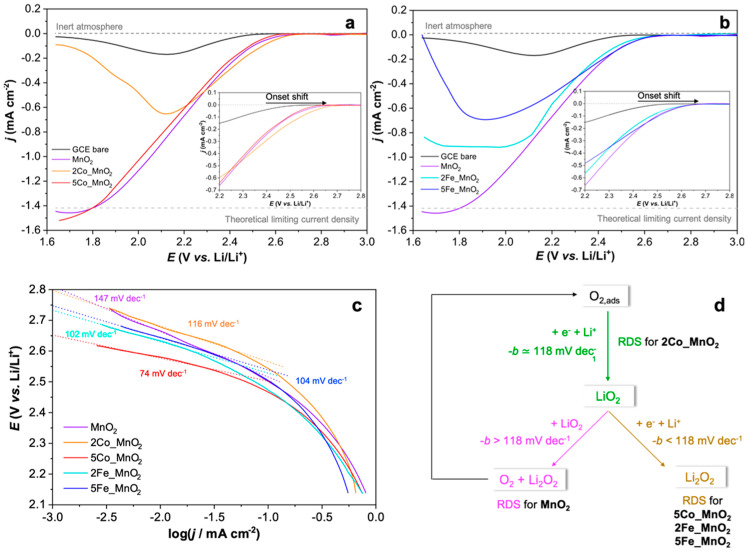
Comparison of staircase–linear sweep voltammetry (S-LSV) curves relative to either bare glassy carbon electrode (GCE) or modified GCE with (**a**) Co-doped and (**b**) Fe-doped MnO_2_. Insets: magnification of the onset zone highlighting the potential shift. S-LSVs were recorded in PC/0.15 M LiNO_3_, by applying a step potential of 50 mV for 10 s (scan rate of 5 mV s^−1^, the current was recorded at the end of each potential step); (**c**) comparison of Tafel slopes (-*b*) relative to GCE-MnO_2_ nano-electrocatalysts evaluated in the 2.45–2.75 V (vs. Li/Li^+^) potential range starting from recorded S-LSVs [37]. Corresponding error values are <2% computed on three measurements; (**d**) possible oxygen reduction pathways according to the adopted electrocatalyst (RDS—rate determining step) [38].

In order to unveil the doping effect of cobalt and iron species in affecting the ORR electrocatalytic activities of MnO_2_ nanomaterial, staircase–linear sweep voltammetries were recorded adopting modified glassy carbon electrodes. In particular, both onset shifts with respect to that of bare glassy carbon and Tafel slopes were assessed, accordingly (Figure 1).

Concerning the former parameter, the best performing sample is 2% Co-doped MnO_2_ for which a shift of about 130 mV towards less cathodic values with respect to bare GCE was obtained (insets of Figure 1a,b; Table 1). Interestingly, by increasing the cobalt (II) content, a significant shift reduction to a value similar to that of pristine MnO_2_ (~50 mV) was observed (inset of Figure 1a; Table 1). Changing the doping species to iron (III) cations, a slight rise of the shift up to 70–80 mV was recorded, even if no remarkable differences were noticed by increasing the dopant amount (inset of Figure 1b; Table 1). Moreover, widening the potential window between 1.6 and 3.0 V vs. Li^+^/Li (Figure 1a,b), only with pure manganese dioxide and 5% Co the theoretical limiting current density was reached, whereas the other electrocatalysts suffer from diffusive limitations. Despite this fact, 2Fe_MnO_2_ achieved a lower limiting current evidencing a smoothly slower kinetics with respect to both pristine and 5% Co-doped powders. This fact can be ascribable to a more favorable poisoning of the active materials pores probably due to the adsorption of the oxygen reduction products [39,40]. Nevertheless, the obtained values are fully in compliance with literature data (reported in Table 1), highlighting that 2Co_MnO_2_ can be a potential candidate for the ORR electrocatalysis.

Furthermore, to have a general overview of the examined system, Tafel slope kinetic parameters were determined considering the S-LSVs data in the range 2.45–2.75 V vs. Li^+^/Li (see Figure 1c) [37]. As reported in literature [42,43], the adsorption of oxygen on the surface of MnO_2_-based electrocatalysts mainly occurs in the form of peroxo-species that create a bridge between two Mn adjacent atoms on the surface. Indeed, the bridge adsorption has been demonstrated to be the most energetic stable configuration [42,43]. Therefore, oxygen is adsorbed molecularly on the manganese dioxide surface, disfavoring its dissociation to atomic species. Augustin et al. [38] deeply unraveled the correlation between Tafel slopes and the possible mechanism for the catalyzed oxygen reduction reaction in organic solvent (LiTFSI/DMSO). As Figure 1d clearly exhibits the adsorbed molecular oxygen can be irreversibly reduced to LiO_2_ through a one-electron process. Subsequently, the formation of Li_2_O_2_ can occur through either chemical disproportion of LiO_2_ or a further one-electron reduction of LiO_2_ [44]. Therefore, Tafel values smaller than 118 mV dec^−1^ are attributed to two consecutive one-electron transfers of which the second one, i.e. the reduction of LiO_2_ to Li_2_O_2_, represents the rate determining step (RDS). Conversely, slopes higher than 118 mV dec^−1^ are due to the chemical disproportion of Li_2_O_2_ and O_2_, which could be re-adsorbed onto the catalyst surface. Finally, values close to 118 mV dec^−1^ indicate the formation of Li_2_O_2_ through LiO_2_ step (as RDS one). Notably, only 2% Co-doped MnO_2_ shows a value very close to 118 mV dec^−1^ (Figure 1c) whereas, on one hand, pristine manganese dioxide has a greater slope, on the other, the remaining three catalysts exhibit a smaller value.

### 3.2. Nanoelectrocatalysts Physicochemical Features

A better understanding of the morphologic, structural and surface properties of the synthesized nanoelectrocatalysts is fundamental to delineate which are the ad hoc features an ideal catalyst for aprotic Li-air batteries should exhibit. Hence, several physicochemical characterizations were carried out.

**Figure 2:**
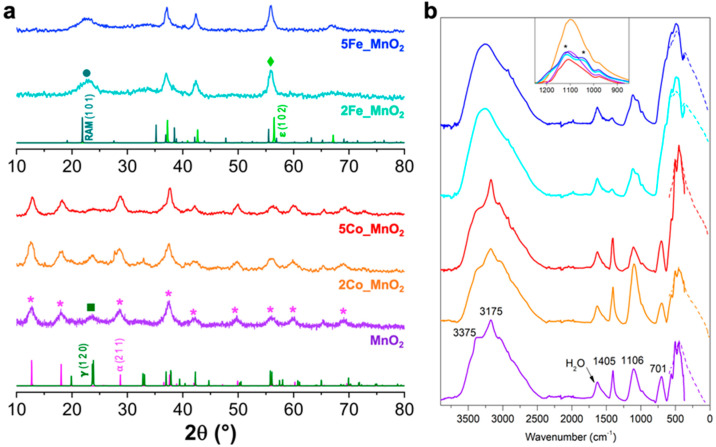
(**a**) Comparison of XRPD patterns relative to bare MnO_2_, Co- and Fe-doped powders. X-ray lines of α-, γ-, ramsdellite and ε-polymorphs are also reported together with the main diffraction peaks and the corresponding Miller’s indices; (**b**) mid- and far-infrared spectra (FIR) for MnO_2_ samples.

On the structural point of view, X-ray diffraction lines (XRPD) show two distinct polymorphic composition for Co- and Fe-doped manganese dioxide nanoparticles (Figure 2a). Specifically, both pure MnO_2_ and Co-MnO_2_ samples are mainly composed by hollandite (α; International Center for Diffraction Data Powder Diffraction File (ICDD PDF-2) Card No. 44-0141 [14]), as the principal phase and nsutite (γ; ICDD PDF-2 n. 14-0644 [45]) MnO_2_. Remarkably, as the dopant content increases, γ-MnO_2_ tends to slightly decrease (5Co_MnO_2_, red pattern in Figure 2a) since its most intense reflection (120) at 2θ of ~24° seems to disappear.

Conversely, the doping with iron species led to a drastic distortion of the MnO_2_ crystal lattice since the aforementioned polymorphic composition was replaced by other two phases, namely ε (ICDD PDF-2 n. 30-0820) and a small amount of β-Ramsdellite (RAM; ICDD PDF-2 n. 04-0378 [46]). Notably, it has been widely stated that either γ or ε show very promising electrocatalytic properties [47,48,49,50]; nevertheless, the combination of these two phases with other polymorphs together with the presence of doping agents is still an open field to investigate. Particularly, we hypothesized that the structural differences, observed with the adoption of a different metallic dopant, may be ascribable to the tendency of the Co^2+^ and Fe^3+^ cations in occupying the tunnels as interstitial species or in substituting the Mn^4+^ ions of the crystal lattice. Concerning cobalt-doped samples, only a smooth difference was observed and this is fully in agreement with literature data that show cobalt doping does not change the main phase structure of MnO_2_ [21,51]. As reported by Peng et al. [51], α-MnO_2_ is characterized by a (2 × 2) tunnel structure in which the large channels are occupied by ammonium cations deriving from the oxidizing agent ((NH_4_)_2_S_2_O_8_) used in the synthetic route. Upon doping with cobalt cations, these NH_4_^+^ species can be substituted by Co^2+^, obtaining a very stable structure as well. On the contrary, the phase transformation process and growth mechanism of Fe-doped MnO_2_ is completely different. Indeed, at an early stage, Fe^3+^ ions occupy the channels of α-MnO_2_ preferentially as interstitial atoms capable of bonding the defective MnO_x_ lattice. Then, other Fe^3+^ accesses the slightly deformed tunnels and continues the bonding process until the (2 × 2) structure disappears. Specifically, due to the continuous collapse and recombination of the structure (i.e., Ostwald ripening mechanism), α-MnO_2_ gradually converts into ε one, as already described by Wang et al. [48]. Finally, most Fe^3+^ substitute Mn^4+^ in the ε polymorph due to the similar hydrated ionic radii (66 pm for iron [52] and 67 pm for manganese [53], respectively). Remarkably, with the Fe-doping, the interplanar distances of ε-MnO_2_ seem to rise and the crystallinity degree decreases, due to both the higher content of lattice defects and oxygen vacancies and the possible presence of a segregated phase (as further corroborated by X-ray photoelectron spectroscopy, XPS and field emission gun-scanning electron microscopy, FEG-SEM images).

In order to shed some light on the peculiar functionalities present in the examined samples, we resorted FTIR spectroscopy: in Figure 2b the spectral features obtained either in ATR mode or in the far infrared are reported with the aim of inspecting as deep as possible these systems. As for the pristine MnO_2_ powder (see the violet curve), a certain number of spectral components are evident along the whole IR range. In particular:

*i.*  in the high wavenumbers range (3600–2800 cm^−1^) a complex envelope is observable, in which some specific contributions can be singled out. The main feature is represented by the band centered at ~3170 cm^−1^, ascribable to the N–H stretching mode of ammonium ions [54] still present in this system (and probably located in the channels of the α phase) and possibly deriving as a residue from the preparation route (see the Materials and Methods section). Moreover, there are two less intense components, located at ~3380 and 3480 cm^−1,^ respectively ascribable, on the basis of their spectroscopic behavior and literature data [55], to the O–H stretching vibrations of water molecules and hydroxyl species located in MnO_6_ octahedra in different coordination positions. Finally, all these bands are superimposed to a large envelope, covering the whole spectral range, which is due to surface OH species mutually interacting by H-bonding;
*ii.*  in the 1700–850 cm^−1^ spectral range at least three more components are evident: the first band, large and located at ~1630 cm^−1^, can be ascribed to the bending mode of undissociated water molecules (whose stretching mode was described above). Then, a sharp component located at ~1400 cm^−1^ can be related to the bending modes of ammonium ions [54,56]. It is worth noting that in the same spectral range lies the S=O asymmetric mode of sulfate species (still deriving from the same oxidizing agent). The third broad component is centered at ~1100 cm^−1^ and presents a satellite shoulder at ~980 cm^−1^: on the basis of the literature, it may be ascribed to the bending mode of OH groups directly bonded to the MnO_6_ octahedra [57]. The shoulder may indicate the possible presence of distorted crystallographic situations on top of which OH groups are still present and exhibit a slightly different spectral position. In this broad envelope, the symmetric S=O stretching mode of residual sulfate-containing species (most likely sulfates deriving from the decompositions of the oxidizing agent employed in the preparation route and whose asymmetric mode is centered at ~1400 cm^−1^, as previously reported) can be appreciable;
*iii.*  last, but not least, in the spectral range below 800 cm^−1^ many components can be observed: in particular, the most intense and broad band at ~690 cm^−1^ ascribable to Mn–O–Mn stretching mode appears, whereas the components located at lower frequency are supposed to be the bending and wagging spectral counterparts of the above species. The other less intense peaks, at ~560, ~500 and ~450 cm^−1^ referred to Mn–O stretching, bending and wagging vibrations from MnO_6_ octahedral units which are shared by corners and/or edges [57,58].

When Co species are added as promoting agents, the main spectral features of MnO_2_ described so far remain almost unchanged (orange and red curves in Figure 2b, respectively due to 2% and 5% Co). The main difference concerns the almost total attenuation of the two shoulders located at very high wavenumbers (at ~3380 and ~3480 cm^−1^) and due to the O–H stretching vibrations of hydroxyl species located in MnO_6_ octahedra in different coordination positions (see point *i* above). This may be correlated to the presence of a Co-containing extra species on the external part of the MnO_6_ octahedral units, thus inhibiting those peculiar vibrational modes. On the contrary, in the case of the promotion by Fe species, some specific differences are evident (bluish curves in Figure 2b, respectively due to 2% and 5% Fe). First of all, the main peak in the high frequency region (at ~3170 cm, the N–H stretching mode of ammonium ions [54]) seems to be almost totally absent as well as the two shoulders discussed so far. This fact may be due to an almost total substitution of NH_4_^+^ ions inside the MnO_6_ octahedral channels by Fe species, leading to a modification of the crystalline structure. Summarizing for the high wavenumbers region, the curve is dominated by a very broad envelope ascribable to the O–H stretching (the spectroscopic bending counterpart is at ~1630 cm^−1^) present at the surface of the Fe-promoted system. As for the region below 1200 cm^−1^, spectroscopically speaking, some interesting features can be evidenced: the broad envelope centered at ~1100 cm^−1^ in the case of pure MnO_2_, becomes more structured, as well as in the presence of Fe species at least 2 components can be singled out (see the inset to Figure 2b). These peaks can still be related to the bending mode of OH groups directly bonded to the MnO_6_ octahedra, in which some positions are now occupied by Fe species, instead by Mn ones, leading to slightly different absorption bands. Far-infrared (FIR) spectroscopy, used for low frequency vibrations, was performed to corroborate the structural features unravel by XRPD analysis. The infrared bands of inorganic materials, as MnO_2_, are generally broad and appear at low wavenumbers. For this reason, FIR is considered an excellent technique to qualitatively study the structural compositions of this type of materials [59,60]. In Figure 2b, FIR spectra for bare and Co- or Fe-doped MnO_2_ nanopowders are reported. Notably, no appreciable differences among FIR curves of pure and cobalt-doped samples can be noticed, confirming the presence of both the α- and γ-phases. Concerning the former, the effective accomplishment of hollandite polymorph was obtained by the appearance of the two most intense bands at ~450 and 500 cm^−1^ [59,61]. γ-MnO_2_, instead, could be described as an irregular intergrowth of elements of ramsdellite and β-pyrolusite (PYR) MnO_2_. In the 300–400 cm^−1^ spectral range, the IR bands are ramsdellite-like, whereas in the 650–700 cm^−1^ range the typical vibrational modes of the pyrolusite polymorph occur [59]. For the Fe-promoted systems, the far-infrared region becomes much less structured, as the presence of Fe species seems to bring about more disorder in the crystallographic structure of MnO_2_. FIR spectra of Fe-doped MnO_2_ (bluish lines in Figure 2b) suggest the presence of different polymorphs with respect to the previous samples, as already found by XRD analysis [62]; one peak is evident, whereas RAM-MnO_2_ definitely causes the appearance of the well-defined band at about 360 cm^−1^ [60].

**Figure 3:**
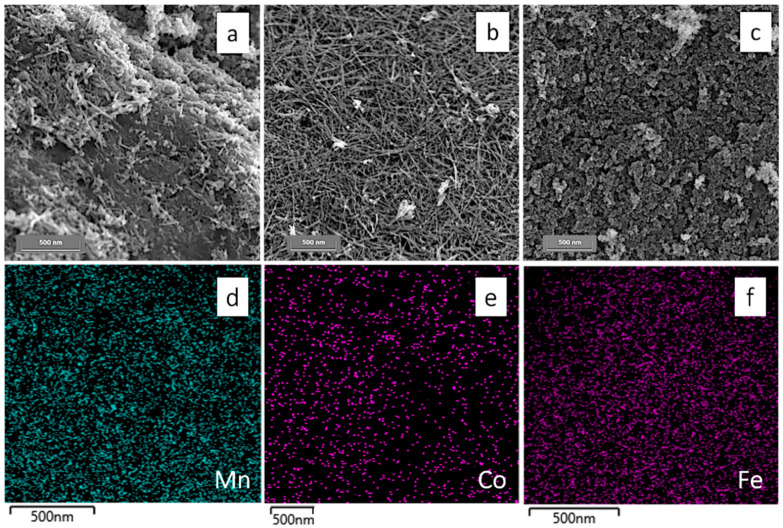
FEG-SEM images of (**a**) bare MnO_2_, (**b**) 5Co_MnO_2_ and (**c**) 5Fe_MnO_2_. EDX mapping relative to (**d**) Mn, (**e**) Co in 5Co_MnO_2_ and (**f**) Fe in 5Fe_MnO_2_ samples.

As far as it concerns the morphological feature of the examined samples, FEG-SEM microscopy has been carried out in order to visualize the nanoparticles surface texture. Figure 3a refers to the bare MnO_2_ system and exhibits acicular particles, with variables length in the range 150–400 nm and a very thin thickness between 20 and 40 nm. Particles of different shape, i.e. more roundish, are seldomly observed: this joint description makes this system very heterogeneous on the morphologic point of view, inhibiting a definite indication of the particles size. On the contrary, when Co is added as doping species (both 2%, Figure S1a and 5%, Figure 3b), an almost unique morphology is evident as the crystallites are composed of thin acicular particles with an extension in length that overcomes that of the bare MnO_2_, being now at least up to 500 nm. A different shape is evident in the case of Fe addition (Figure 3c and Figure S1b); actually, the particles show an almost roundish appearance with an increased tendency to agglomerate.

Moreover, in order to assess the effective dopants, amount together with their possible segregation on MnO_2_ surface and the lattice defectivity, energy dispersive X-ray spectroscopy (EDX) together with X-ray photoelectron spectroscopy (XPS) measurements were performed. Concerning the former (from the mapping reported in Figure 3d–f and Table 2, 2nd column), it can be noted that in the case of the cobalt addition this remains well under the half of the theoretical amount. In addition, Co species seem to be homogeneously distributed indicating no segregation, as evident in Figure 3e.

On the contrary, when Fe species are present, the percentage is pretty much higher that the theoretical one (Table 2, 2nd column) highlighting that, at the same EDX experimental conditions, there is a sort of enrichment in this species in the outer layers of the composite material, giving rise to a possible segregation. Indeed, in the EDX map (Figure 3f), Fe species are densely observable, almost totally covering the MnO_2_ material. All the assumptions reported so far are well in agreement with the results relative to XP analyses.

**Figure 4:**
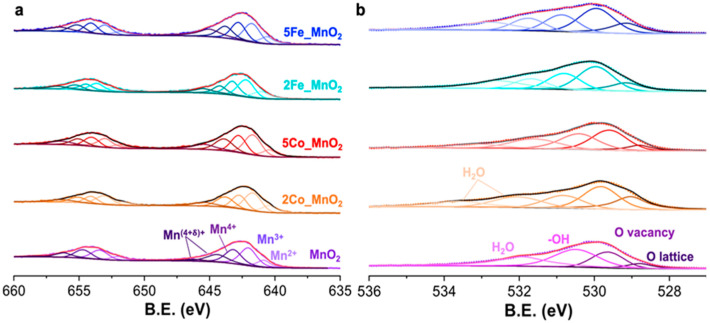
Fitted XP spectra of (**a**) Mn 2p and (**b**) O 1s regions relative to bare, Co- and Fe-doped MnO_2_ samples.

Specifically, regarding the Co or Fe/Mn XPS molar ratios (Table 2, 2nd and 3rd columns), we were able to evaluate only iron doping since the Co 2p XPS region (Figure S2a) falls within the same range of Mn 2s. Instead, for Fe/Mn ratios, we obtained very huge values (11 and 24 atomic percentage, respectively for 2% and 5% doping; Figure S2b) with respect to the expected ones from the synthetic route. This may be caused by the segregation of iron species on the surface of manganese dioxide, thus leading to the possible formation of an iron shell-like structure. Besides, focusing on the Mn 2p and O 1s high-resolution spectra (Figure 4), a corroboration of the previous outcomes concerning the lattice defectivity upon doping was attained. Actually, for either Co– or Fe–MnO_2_ nanoparticles an increment of the Mn species with an oxidation state greater than 4 (i.e., Mn^(4+δ)+^) can be observed (see Figure 4a and Table 2, 4th column). Particularly, this rise is proportional to the dopant amount underlining the possible increase in lattice defects. Precisely, this defectivity was further verified by focusing on the O 1s region (Figure 4b): the peak at ~529.7 eV is reported to be ascribable to high binding energy component (HBEC) developed with the increasing of oxygen vacancies [63,64]. Instead, the other peaks are, respectively correlated to: *(i)* oxygen bound to the metal ions in the lattice (at ~528.8 eV) [14,65]; *(ii)* low binding energy component (LBEC) due to adsorption of OH^−^ on the surface (at ~530.5 eV [63,66,67]); and *(iii)* water adsorption (at B.E. equal or higher than 531.9 eV) [66,68]. Remarkably, for cobalt-doped powders the band relative to oxygen vacancies increases by adding more dopant (Table 2, 5th column); conversely, it seems that after a certain Fe content further vacancy may not be formed. This may be explained once more by the growth of a segregated phase, especially in the 5% Fe_MnO_2_, which may have hindered the additional formation of oxygen vacancies.

**Figure 5:**
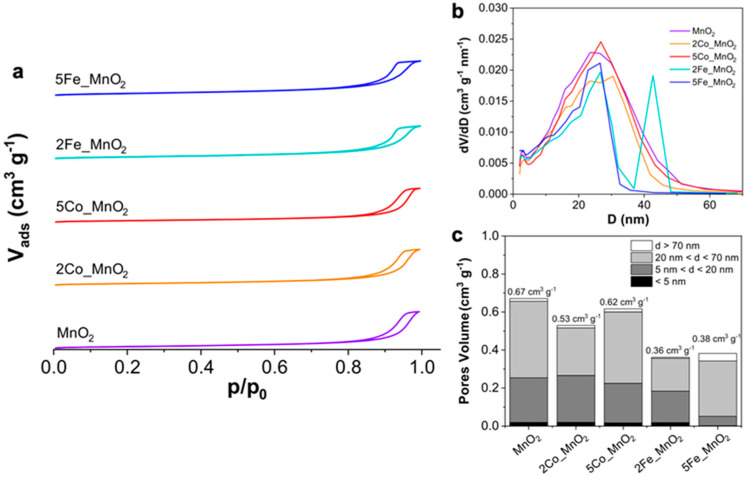
Comparison of (**a**) Brunauer-Emmett-Teller (BET) isotherms and (**b**) Barrett-Joyner-Halenda (BJH) pore-size distribution of all the as-synthesized nanoelectrocatalysts; (**c**) histogram showing the pores volume distribution together with the total pore volume.

Finally, concerning the surface properties, BET-BJH analyses were carried out. For all the synthesized electrocatalysts, BET hysteresis loop (Figure 5a) evidence the presence of bottled-neck pores [69].

Moreover, as clearly stated in Table 2 (6th column), the 2% cobalt doping seems to smoothly reduce the specific surface area (from 122 to 98 m^2^ g^−1^) and the total pore volume (from 0.67 to 0.53 cm^3^ g^−1^; Figure 5b,c), while its further rise reestablishes both these two parameters (118 m^2^ g^−1^ and 0.62 cm^3^ g^−1^) that result very similar to those of pristine MnO_2_. Contrarily, iron species tend to decrease the active surface area (89 and 100 m^2^ g^−1^ for 2 and 5% doping, respectively) and the total pore volume (0.36 and 0.38 cm^3^ g^−1^). However, particularly for 5Fe_MnO_2_, a change in the porosity distribution was observed (Figure 5c) since the number of pores with diameter smaller than 20 nm dramatically lowers.

## 4. Discussion

Once investigated all the nanopowders physicochemical features, we tried to combine them with the observed electrocatalytic behavior. We can infer that the optimal electroactivity is a balance between the desired ORR onset shift with respect to our benchmark (GCE) and the Tafel slopes (electron transfer pathway). As concerns the former parameter, the most performing nanomaterial is 2% Co-doped MnO_2_, due to both the integration of one of the most electrocatalytic MnO_2_ polymorph, namely γ-nsutite, into the α-phase and the higher lattice defectivity as seen by XPS analyses. As such a greater number of active sites was obtained. These hypotheses are further confirmed by studying the 5% Co-doped sample. Indeed, for this compound, γ-phase seems to disappear leading to a worsening of the potential shift value. Conversely, regarding the Fe insertion, either the two iron-doped nanopowders exhibited a similar behavior towards ORR (shift of ~70–80 mV). Once more, we can unveil this observation since the increased amount of Fe only leads to the growth of a shell-like segregated phase. Finally, our investigation also highlighted the possible higher electroactivity of the γ-integrated phase in the α-polymorphic matrix rather than ε one.

Moreover, as far as it regards the kinetic point of view, we witnessed three different behaviors, as clearly evidence in Figure 1d. Particularly, in the case of pristine manganese dioxide, a Tafel slope greater than the theoretical one (118 mV dec^−1^ in aprotic solvents [44]) was reached, indicating the occurrence of the chemical disproportion to form Li_2_O_2_, i.e., the main reduction product in Li-air batteries and O_2_. Furthermore, Li_2_O_2_ is also the desired discharge product since it was observed that it decomposes to Li and O_2_ during the electrochemical charging process and this charge/discharge cycling lasts for many cycles [70]. Notably, since this nanoelectrocatalyst possesses both the highest pores volume and surface area, the pores clogging by lithium peroxide was rather prevented. This is the reason it achieved the theoretical limiting current density displayed in Figure 1a,b. Moreover, it is worth noting the different behavior of Co-doped samples. Only 2Co_MnO_2_ exhibited a Tafel slope very close to the theoretical one, resulting in the formation of LiO_2_ as the RDS. This species is significantly important for the device performances, since its low stabilization by aprotic solvent molecules can lead to the formation of Li_2_O, instead of Li_2_O_2_, that is rather insoluble and can provoke short-circuits [36]. However, also lithium superoxide is less soluble and can cause to a less extent the clogging of the cathode pores [36]. This may explain the difficulty for the 2% Co-electrocatalyst in achieving the theoretical limiting current, since its low mesoporosity can favor the pores clogging. Conversely, 5% Co alongside with iron-doped samples showed slopes smaller than 118 mV dec^−1^, thus being characterized by two consecutive electron transfers of which the second one (formation of Li_2_O_2_) is the RDS. Nevertheless, the only powder that could reach the theoretical limiting current is the 5% Co-MnO_2_. This may be ascribable to both the almost absence of γ-polymorph with its smaller interlayer tunnels (see XRPD in Figure 2a) and the porosity, very similar to pure manganese dioxide one (Figure 5). Furthermore, a reduced limiting current value was also noticed for 2Fe_MnO_2_; whereas the higher Fe amount led to the poisoning of the cathode surface. For the latter, indeed, a too great dopant content resulted in the formation of a segregated phase (as clearly seen from FTIR, FEG-SEM/EDX images and XPS results) which may have hindered the electroactivity of the manganese dioxide nanoparticles.

## 5. Conclusions

Herein, we have tried to unravel the efficacy of either pristine or Co/Fe-doped MnO_2_ in enhancing the electronic properties towards ORR processes. In particular, we evaluated the possible gain in terms of ORR onset shift towards less cathodic values, the rate of the electronic transfer, though the slope of the linear sweep voltammetries and the assessment of the electron transfer pathway by Tafel slopes.

XRPD and IR analyses clearly showed the peculiar polymorphic composition by changing the doping agent, i.e. from a combination of α and γ phases for both pure and Co-doped MnO_2_ to ε and ramsdellite (RAM, as a minority one) polymorphs for Fe-MnO_2_, due to Mn^4+^ gradual substitution by Fe^3+^ ions. In addition, for the latter, an increase of dopant amount led to the formation of a segregated iron oxide shell, as seen by FEG-SEM/EDX and XPS techniques, that may hinder the potential electrocatalytic behavior. Notably, both the active surface area together with the pores dimensions and the lattice defectivity do play a pivotal role in tuning the final electrocatalysts properties. As far as it concerns the ORR onset shift, 2% of cobalt seems to boost the oxygen reduction moving the corresponding potential towards less cathodic values with respect to both pure MnO_2_ and 5% of this dopant inside the manganese dioxide lattice. Indeed, in this latter compound the presence of γ polymorph—which is reported to be highly electroactive—is reduced. However, due to its slightly higher total pore volume and a pores distribution very similar to bare MnO_2_ one, it allows to reach the theoretical limiting current which is not achieved in the case of 2Co_MnO_2_ sample. Actually, according to the hypothesized ORR pathway, a low cobalt content could be characterized by the one-electron transfer reaction to LiO_2_ as RDS (Tafel slope of ~118 mV dec^−1^), thus resulting in the main production of lithium superoxide that could clog the cathode pores. The same reasoning can be drawn for iron-doped materials notwithstanding the changing in the polymorphic composition. In this context, both samples exhibited a Tafel slope smoothly smaller (around 100 mV dec^−1^) than 118 mV dec^−1^ denoting a different RDS, namely the second electron transfer from LiO_2_ to Li_2_O_2_. Furthermore, in this case, the lithium peroxide can clog the cathode pores due to its scarce solubility in PC solvent. Interestingly, the poisoning of the 5% Fe-doped MnO_2_ occurred more quickly probably due to the observed segregated phase, which partially covered the electrocatalyst active sites.

Hence, by combining the observed electrocatalytic activity and the physicochemical features, we succeeded in giving the guidelines to tailor the MnO_2_ synthetic route in order to prepare promising low-cost nanoelectrocatalysts, that have boosted surface features and lattice defectivity. These two parameters revealed fundamental for the design of very active nanomaterials without diffusive limitations.

## Figures and Tables

**Table 1:** Comparison of oxygen reduction reaction (ORR) onset potential shifts obtained using different electrocatalysts deposited on GCE, from either literature or this work. For the latter, standard deviations computed on at least 3 measurements are reported. Onset potentials shifts were computed considering bare GCE as benchmark. GCE onset = 2.59 V vs. Li/Li^+^.

Catalysts	Solvent/Electrolyte	ORR Onset Shift (mV)	Ref.
Pt/C	LiClO_4_/DME	30	[41]
Pd/C		0	
Au/C		50	
Pt/C	LiClO_4_/TEGDME	70	
Pd/C		90	
Au/C		130	
Pt/C	LiClO_4_/DMSO	60	
Pd/C		60	
Au/C		50	
Carbon vulcan XC72R	LiTFSi/DMSO	20	[38]
Mn_5_O_8_/C		20	
α-Mn_2_O_3_/C		110	
MnO_2_	0.15-M LiNO_3_/PC	50 ± 2	This work
2Co_MnO_2_		130 ± 5	
5Co_MnO_2_		60 ± 3	
2Fe_MnO_2_		70 ± 2	
5Fe_MnO_2_		80 ± 5	

**Table 2:** Estimation of dopant/Mn molar ratios by energy dispersive X-ray spectroscopy (EDX) and X-ray photoelectron spectroscopy (XPS). XPS ratios between counts of Mn^(4+δ)+^ (relative to Mn 2p^3/2^) or oxygen vacancy (O_vacancy_) peaks and total counts for all the investigated samples. BET specific surface area (*S*_BET_). n.d. = no data.

Sample	% Co or Fe/Mn		Mn^(4+δ)+^/Mn_tot_	O_vacancy_/O_tot_	*S*_BET_ (m^2^ g^−1^)
	EDX	XPS			
MnO_2_	−	−	0.26	0.22	122
2Co_MnO_2_	0.6	n.d.	0.32	0.30	98
5Co_MnO_2_	2.0	n.d.	0.36	0.39	118
2Fe_MnO_2_	7.7	11	0.28	0.32	89
5Fe_MnO_2_	11.0	24	0.39	0.32	100

## Supplementary Materials

The following are available online at https://www.mdpi.com/2079-4991/10/9/1735/s1, Figure S1: FEG-SEM images of (**a**) 2Co_MnO_2_ and (**b**) 2Fe_MnO_2_; Figure S2: XP spectra of (**a**) Co 2p and (**b**) Fe 2p regions relative to 5Co_MnO_2_ and 5Fe_MnO_2_, respectively.
