# Supplementary material for: ORR in Non-Aqueous Solvent for Li-Air Batteries: The Influence of Doped MnO2-Nanoelectrocatalyst
Source: Nanomaterials (Basel). 2020 Sep 1;10(9):1735. doi: 10.3390/nano10091735 (PMC7558571; doi:10.3390/nano10091735)
Supplement: Supplementary file 1 [file nanomaterials-10-01735-s001.pdf]

Supplementary Information

ORR in Non-Aqueous Solvent for Li-Air Batteries: the Influence of Doped MnO_2-Nanoelectrocatalyst

Eleonora Pargoletti [1,2], Annalisa Salvi [1], Alessia Giordana [3], Giuseppina Cerrato [3], Mariangela Longhi [1,2], Alessandro Minguzzi [1,2], Giuseppe Cappelletti [1,2,*] and Alberto Vertova [1,2,*]

[1] Dipartimento di Chimica, Università degli Studi di Milano, Via Golgi 19, 20133 Milan, Italy; eleonora.pargoletti@unimi.it (E.P.), annalisa.salvi@studenti.unimi.it (A.S.), mariangela.longhi@unimi.it (M.L.), alessandro.minguzzi@unimi.it (A.M.)

[2] Consorzio Interuniversitario per la Scienza e Tecnologia dei Materiali (INSTM), Via Giusti 9, 50121, Firenze, Italy

[3] Dipartimento di Chimica & NIS, Università degli Studi di Torino, Via P. Giuria 7, 10125, Turin, Italy; giuseppina.cerrato@unito.it (G.Ce.), alessia.giordana@unito.it (A.G.)

* Correspondence: giuseppe.cappelletti@unimi.it (G.C.) and alberto.vertova@unimi.it (A.V.); Tel.: +39-02-50314228 (G.C.); +39-02-50314232 (A.V.)

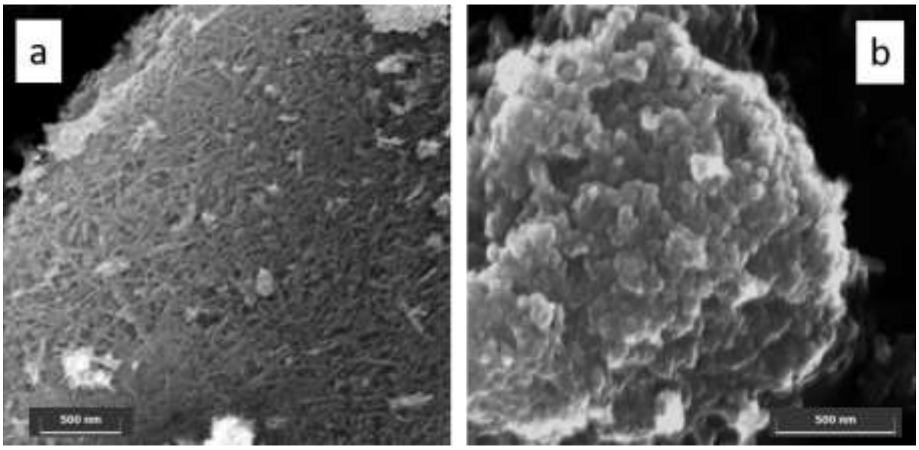

Figure 1. FEG-SEM images of (**a**) 2Co_MnO_2 and (**b**) 2Fe_MnO_2.

For the sake of clarity, a couple of FEG-SEM images referred to the 2% promoted materials was represented in Figure S1, in order to confirm the role of the dopants in modifying the final surface texture of the samples. The same trend of 5% doped MnO_2 was observed for lower dopants amount, either Co or Fe, since the system promoted by 2% Co still exhibits acicular shape of the particles (see Figure S1a) with the same characteristics of the 5% Co sample (Figure 3b). Analogously, the 2% Fe-doped MnO_2 behaves likewise to its 5% counterpart, as its particles show totally different features (see Figure 3c) if compared to the bare MnO_2: a low amount of Fe species leads to almost roundish shape with a fast tendency to aggregate (see Figure S1b).

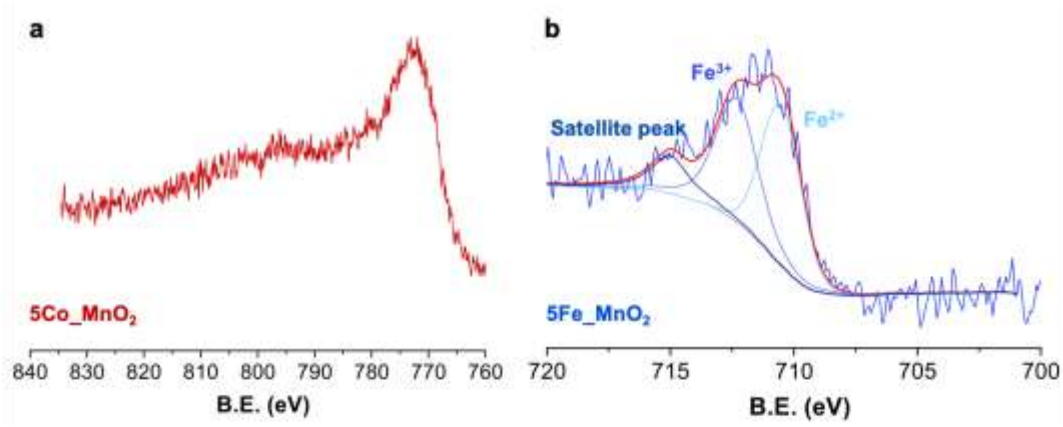

Figure S2. XP spectra of (**a**) Co 2p and (**b**) Fe 2p regions relative to 5Co_MnO_2 and 5Fe_MnO_2 respectively. The Co 2p region was not fitted since it falls within the same range of Mn 2s. Besides, the Fe high-resolution spectrum was deconvoluted into three peaks ascribable to Fe^{2+}, Fe^{3+} species and a satellite one, according to Biesinger *et al.* [65].
